# Regulatory T-Cells in Chronic Lymphocytic Leukemia and Autoimmune Diseases

**DOI:** 10.4084/MJHID.2012.053

**Published:** 2012-08-09

**Authors:** Giovanni D’Arena, Giovanni Rossi, Barbara Vannata, Silvia Deaglio, Giovanna Mansueto, Fiorella D’Auria, Teodora Statuto, Vittorio Simeon, Laura De Martino, Aurelio Marandino, Giovanni Del Poeta8, Vincenzo De Feo, Pellegrino Musto

**Affiliations:** 1 Department of Onco-Hematology, IRCCS “Centro di Riferimento Oncologico della Basilicata”, Rionero in Vulture, Italy; 2 Hematology and Stem Cell Transplantation Unit, IRCCS “Casa Sollievo della Sofferenza”, San Giovanni Rotondo, Italy; 3 Hematology Institute, Catholic University of “Sacred Hearth”, Rome, Italy; 4 Human Genetics Foundation (HuGeF) and Laboratory of Immunognenetics, University of Turin, Italy; 5 Laboratory of Preclinical and Translational Research, IRCCS “Centro di Riferimento Oncologico della Basilicata”, Rionero in Vulture, Italy; 6 Department of Pharmaceutical and Biomedical Sciences, University of Salerno, Italy; 7 Hematology Institute, “Tor Vergata” University, Rome, Italy

## Abstract

Regulatory T-cells (Tregs) constitute a small subset of cells that are actively involved in maintaining self-tolerance, in immune homeostasis and in antitumor immunity. They are thought to play a significant role in the progression of cancer and are generally increased in patient with chronic lymphocytic leukemia (CLL). Their number correlates with more aggressive disease status and is predictive of the time to treatment, as well. Moreover, it is now clear that dysregulation in Tregs cell frequency and/or function may result in a plethora of autoimmune diseases, including multiple sclerosis, type 1 diabetes mellitus, myasthenia gravis, systemic lupus erythematosus, autoimmune lymphoproliferative disorders, rheumatoid arthritis, and psoriasis. Efforts are made aiming to develop approaches to deplete Tregs or inhibit their function in cancer and autoimmune disorders, as well.

## A Brief History

The human immune system is a well-coordinated network of cells, organs and glands acting in harmony to protect the host from a broad range of pathogenic microorganisms and, at the same time, to avoid responsiveness to self-antigens (*immunological self-tolerance*) and to control the quality and the magnitude of immune responses to non-self-antigens thus avoiding damage to the host (*immune homeostasis*). Several mechanisms are thought to be involved in this complex control system (Table 1). In this scenario, a distinct small subset of specialized T-lymphocytes, the so-called regulatory T-cells (Tregs), seem to play a pivotal role in maintaining homeostasis and self-tolerance.^1^,^2^ In fact, Tregs act suppressing the function of self-reactive T-cells to protect the host from autoimmune disease. At the same time they seem to be able to prevent antitumor immune responses.^3^

Gershon and Kondo of Yale University firstly proposed the existence of T-cells with suppressive activity more than 40 years ago.^4^ However, its better identification lacked for several years and this field of research shrank until to 1995, when Shimon Sakaguchi and coworkers identified a population of CD4^+^ T-cells expressing surface interleukin-2 (IL)-2 receptor α-chain (recognized by CD25) and termed them ‘regulatory’ T-cells.^5^ However, CD25 is not exclusively restricted to Tregs because of its expression on the surface of T effector lymphocytes after activation.^6^ Baecher-Allan and co-workers, by means of flow cytometry and *in vitro* study of sorted cells, identified a very small subset of T cells with high expression of CD25 that exhibited a strong regulatory function in humans.^7^–^9^ CD4^+^CD25^+high^ cells inhibited proliferation and cytokine secretion by activated CD4^+^CD25^+^ responder T-cells in a contact-dependent manner.

In addition, it has been experimentally demonstrated that depleting Tregs produces inflammatory bowel disease, resulting from excessive immune response to intestinal commensal bacteria.^10^ Finally, reducing or removing Tregs leads to effective tumor immunity leading in turn to tumor eradication.^11^,^12^

More recently, the intracellular transcription factor forkhead/winged helix box P3 (FoxP3), also called scurfin, has been identified as the most accepted marker for Tregs.^13^–^15^ It functions regulating a set of genes involved in the suppression, proliferation and metabolic activities of Tregs. Moreover, CD127, that identified the heterodimeric IL-7 receptor, combined with CD4, CD25 and FoxP3, has been shown to better identify Tregs avoiding the contamination of this small cell population (accounting for 1–4% of circulating CD4^+^ lymphocytes in humans) with activated T-cells.^16^,^17^

## Tregs and Autoimmunity

It is now clear that dysregulation in Tregs cells may result in a plethora of autoimmune diseases, including multiple sclerosis, type 1 diabetes mellitus, myasthenia gravis, systemic lupus erythematosus, autoimmune lymphoproliferative disorders, rheumatoid arthritis, and psoriasis.^18^

As a matter of the fact, complex genetic disorders typically associated with the MHC chromosomal region as well as the dysregulation of Treg cells frequency and/or function appear to be involved in autoimmune diseases.^19^ In particular, FoxP3, IL-2 and relative receptor play a key role in the maintenance of Tregs associated pathological immune responses.^20^

Deficiency in FoxP3 due to genetic mutations results in a lethal X-linked recessive lymphoproliferative disease in mice and human subjects characterized by immunodysregulation, polyendocrinopathy, enteropathy, X-linked (IPEX) syndrome.^21^ This autoimmune disorder is characterized by a severe intestinal pathology, with massive T-cell infiltration, type 1 diabetes mellitus, eczema, anemia, liver infiltration, thrombocytopenia, hypothyroidism, and the presence of various autoantibodies. FoxP3 deficiency was also found in the multiple sclerosis although Treg cells frequency was comparable with healthy individuals.^22^,^23^ Similar results emerged in type 1 autoimmune diabetes, psoriasis, myasthenia gravis and autoimmune polyglandular syndromes (APS).^24^–^26^ The degree of deficiency of functional anomaly of FoxP3^+^ natural Tregs is able to alter the manifestation of autoimmunity. Alterations of Tregs were also reported in rheumathoid arthtritis and in idiopathic juvenile arthritis. Results obtained may suggest a possible role of Tregs in the downregulation of the joint inflammation.^27^

## Defining Tregs

**Figure 1:**
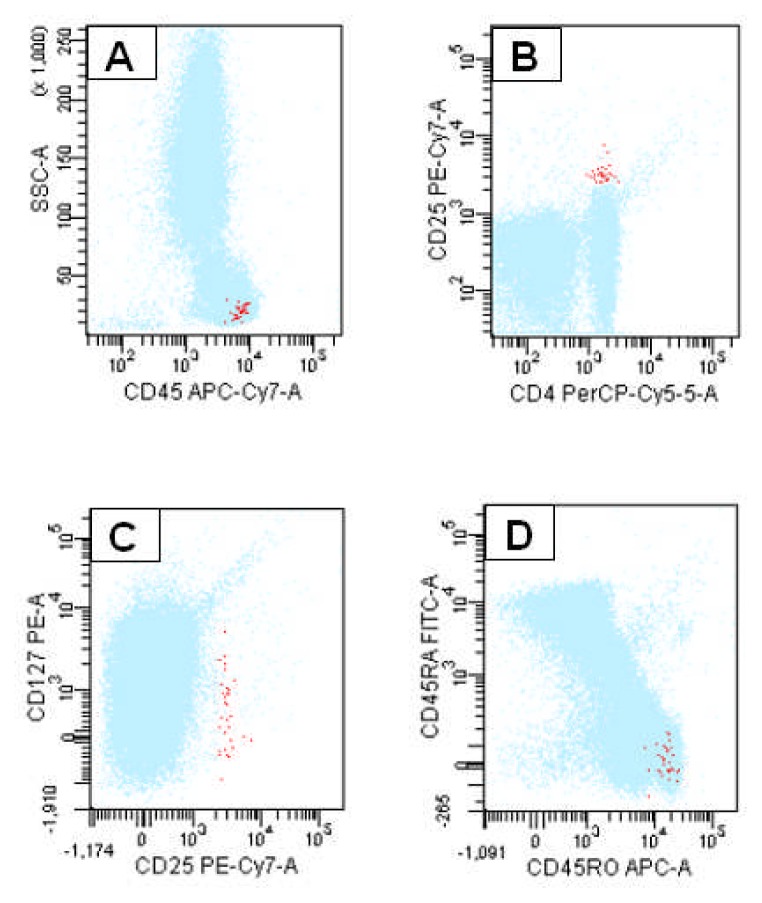
Flow cytometric detection of Tregs. Tregs are CD4+ lymphocytes displaying a CD45 expression of T-cell subpopulations (A). CD25 antigen is expressed at high density whereas CD127 at low to undetectable levels (B and C).Selected CD25+/CD127+ lymphocytes are positive for CD45RO (D).

Taken all above into account, Tregs may be defined as a small population of T-cells with a relevant role in the immune homeostasis. For this reason, they are actively involved in the immunosurveillance against autoimmune disorders and cancer, as well. Tregs may be defined as CD4^+^ T-cells expressing CD25 at high levels, cytoplasmic FoxP3, and very low to undetectable CD127 on their surface (Figure 1). However, several other markers have been associated to Tregs, but none of them may be considered as a unique marker (Table 2).

**Figure 2:**
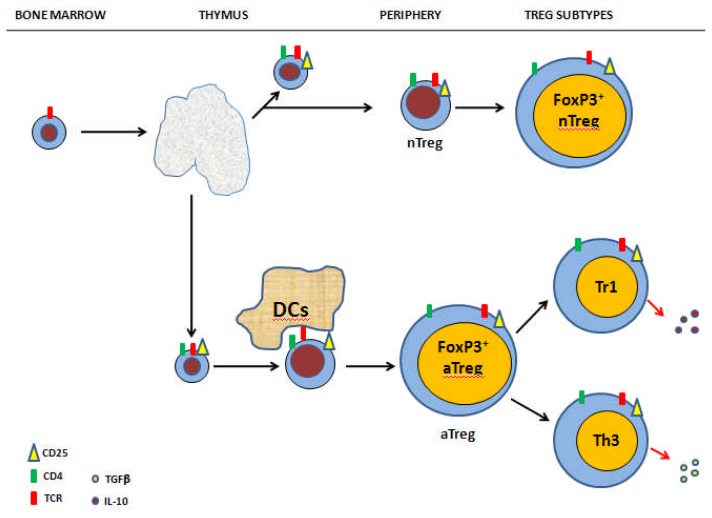
Regulatory T-cells: development and subsets. Three major subjects of Tregs have been recognized so far. A) Tregs (innate and adaptative): they express CD25, FoxP3, CTLA-4, αβ-TCR, and secrete the immunosuppressive lymphokines IL-10 and TGF-β. B) Tr1 cells: they do not express FoxP3 nor large amount of CD25, secrete IL-10 and TGF-β. Tr1 cells are abundant in the intestine where they elicit their main function that is making tolerance to the many agents that are part of its diet. C) Th_3_ cells: they are also prevalent in the intestine and like to Tr1 cells act suppressing immune responses to ingested antigens (oral tolerance) by means of TGF-β secretion.

Two main subsets of Tregs have been described according to their origin. *Innate (or naturally occurring)* Tregs originate in the thymus as a consequence of the interaction with high-affinity antigens expressed in thymic stroma and constitutively expressing FoxP3.^28^ They are involved in immune homeostatis, thus suppressing the response against self antigens. Such cells persist throughout life despite thymic involution after puberty. *Adaptative* Tregs emerges also from the thymus but acquire its suppressive activity in periphery regulating the response against self and non-self-antigens.^29^ Figure 2 summarizes the generation and subpopulations of Tregs.

Tregs have been shown to suppress the proliferation of antigen-stimulated naïve T-cells and several mechanisms have been suggested by means of which they elicit their suppressive activity.^30^,^31^ Either natural and adaptative Tregs are antigen-specific and are seen to need T-cell receptor (TCR) triggering to become suppressive^31^,^32^ despite this latter point is still controversial.^33^*, In vitro* studies suggested that activated Tregs suppress activated CD4^+^ or CD8^+^ effector T-cells by means of cell-to-cell contact. In this mechanism a crucial role is played by the ligation of CD80/CD86 complex on effector cells by cytotoxic T-lymphocytes antigen-4 (CTLA-4) on Tregs surface resulting in the transmission of inhibitory signals of T-cell function.^34^,^35^ In a similar fashion, Tregs seem to modulate dendritic cells (DCs) function resulting in the expression and activation of indoleamine 2,3-dioxygenase degradation.^36^ DCs may be blocked in maturation and/or activation by release of IL-10 and TGF-β that resulting in antigen-presenting capacity impairment due to down-regulation of major histocompatibility complex (MHC) class II and in interfering in costimulatory molecules expression.^37^,^38^ Other *in vitro* studies suggest Tregs inhibition by means of the release of suppressive cytokines, such as IL-10 and TGF-β.^39^–^41^ Activated Tregs are capable to express granzyme A or perforin and kill activated CD4^+^ or CD8^+^ T-cells, through the perforin-dependent way.^42^,^43^

## Tregs and Chronic Lymphocytic Leukemia

Chronic lymphocytic leukemia (CLL), the most common form of leukemia in Western countries, is characterized by the accumulation of monoclonal B-lymphocytes in bone marrow, lymphoid organs and peripheral blood.^44^ Moreover, there is increasing evidence of T cell dysfunction in CLL and this may probably contribute to the etiology and the progression of the disease.^45^,^46^ Several authors reported that Tregs are increased in CLL patients.^47^–^51^ Using multicolor flow cytometry, we showed that CLL patients had a higher absolute number of circulating Tregs compared to age and sex-matched controls.^51^ In addition, Tregs cell number was significantly correlated to more advanced Rai clinical stages, peripheral blood B-lymphocytosis, more elevated LDH levels, and absolute number of CD38^+^ neoplastic B-cells.

The evidence that Tregs are reduced after therapy with fludarabine, agrees with the hypothesis that these cells play a critical role in protecting CLL cells from getting killed by the immune system.^47^ The same happens when patients with CLL were treated with thalidomide.^52^ This drug and its analogues, such as lenalidomide, acts as immunomodulatory agents targeting the microenvironment and both are shown to be effective in the treatment of CLL patients, probably by means of TNF modulation.^53^–^55^

**Figure 3:**
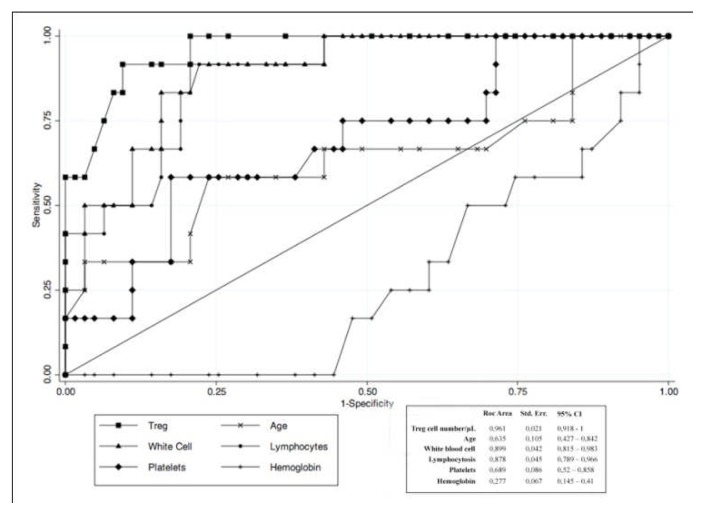
ROC curve graphically showing the trade-offs between sensitivity and specificity for different cutoffs used to discriminate between positive and negative cases (i.e., treatment demand vs no treatment demand patients). The best predictive cutoff of circulating Treg cell number seems to be in the range from ≥40 to ≥42/μL. The result of cutoff ≥41/μL shows the best predictive power among the others.

The prognostic role of Tregs have been poorly investigated. Only two paper reported that a shorter time to first treatment may be predicted by the circulating number of Tregs.^56^,^57^ As showed in Figure 3, we found a best predictive cut-off of absolute circulating Tregs able to identify patients with early stage CLL at higher risk of requiring therapy.^57^

**Figure 4:**
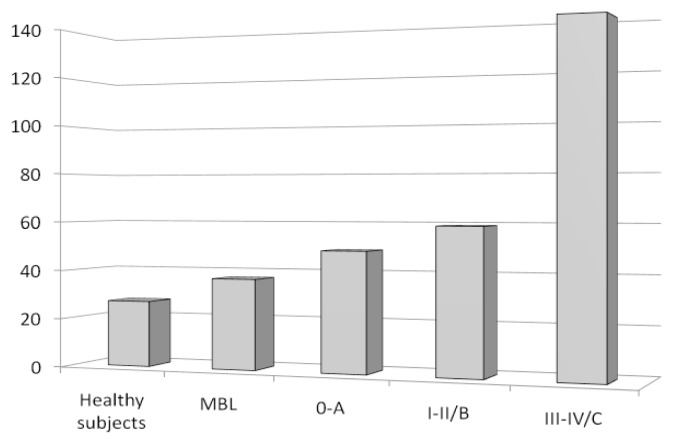
Circulating Tregs number in healthy subjects, MBL and CLL patients grouped according to Rai/Binet clinical stages. Data are expressed as mean absolute circulating Tregs number (/μL) ± standard deviations.

Finally, we have studied Tregs in ‘clinical’ monoclonal B-cell lymphocytosis (MBL), a condition in which less than 5000/μL circulating monoclonal B-cells, in absence of other features of lymphoproliferative disorders, is found.^58^ We showed that MBL patients had a lower absolute number of Tregs, compared to CLL patients, but higher than controls (Figure 4).^59^ Taken together, these data show that the tumor mass (from MBL low to intermediate to high-risk CLL) and the circulating Tregs increase simultaneously, thus suggesting that the expected result is a more robust inhibition of tumor inhibiting cells and, ultimately, a greater expansion of neoplastic B cells.

## Conclusions

Tregs play a critical role in immune tolerance (maintaining peripheral tolerance to self-antigens) and in immune homeostasis (regulating the immune response to non self-antigens). Moreover, it is now clear that Tregs have a role in suppressing tumor-specific immunity and for that reason are actively involved in the etiology and in progression of cancer, such as CLL, the most frequent form of leukemia in Western countries. Tregs disregulation is thought to be also involved in the pathogenesis of autoimmune disorders. In light of this, Tregs appear as having a great potential in treating autoimmunity and cancer. There is now considerable evidence in preclinical models to suggest that adoptive Tregs therapy will be highly efficacious. For that reason, clinical strategies are developing to target such cells aiming to modulate their suppressive function.^60^–^65^

## Figures and Tables

**Table 1:** The main mechanisms of immunological tolerance

**Central tolerance**	Clonal deletion
	Clonal anergy
	Receptor editing
**Peripheral tolerance**	Immune deviation
	Suppression
	Immune privilege
	Network-mediated regulation
	Coreceptor modulation

**Table 2:** Immunophenotype of Tregs

Antigen	Expression
CD4	Positive
CD8	Negative
CD25	High
CD127	Low to undetectable
FoxP3	Positive
GITR	High
IL-10	Positive
TGF-β	Positive
CD152 (CTLA-4)	High
CD154 (CD40L)	Negative
CD45RA	Negative
CD45RO	Positive
